# Triptolide increases resistance to bile duct ligation-induced liver injury and fibrosis in mice by inhibiting RELB

**DOI:** 10.3389/fnut.2022.1032722

**Published:** 2022-10-13

**Authors:** Zihang Yuan, Jie Wang, Haoran Zhang, Yingying Miao, Qianhui Tang, Ziqiao Yuan, Cheng Nong, Zhicheng Duan, Luyong Zhang, Zhenzhou Jiang, Qinwei Yu

**Affiliations:** ^1^New Drug Screening Center, Jiangsu Center for Pharmacodynamics Research and Evaluation, State Key Laboratory of Natural Medicines, China Pharmaceutical University, Nanjing, China; ^2^Center for Drug Research and Development, Guangdong Pharmaceutical University, Guangzhou, China; ^3^Key Laboratory of Drug Quality Control and Pharmacovigilance, Ministry of Education, China Pharmaceutical University, Nanjing, China

**Keywords:** RelB, bile duct ligation (BDL), triptolide, TNFSF14, cholangiocyte

## Abstract

Cholestasis is a common, chronic liver disease that may cause fibrosis and cirrhosis. *Tripterygium wilfordii* Hook.f (TWHF) is a species in the Euonymus family that is commonly used as a source of medicine and food in Eastern and Southern China. Triptolide (TP) is an epoxy diterpene lactone of TWHF, as well as the main active ingredient in TWHF. Here, we used a mouse model of common bile duct ligation (BDL) cholestasis, along with cultured human intrahepatic biliary epithelial cells, to explore whether TP can relieve cholestasis. Compared with the control treatment, TP at a dose of 70 or 140 μg/kg reduced the serum levels of the liver enzymes alanine transaminase, aspartate aminotransferase, and alkaline phosphatase in mice; hematoxylin and eosin staining also showed that TP reduced necrosis in tissues. Both *in vitro* and *in vivo* analyses revealed that TP inhibited cholangiocyte proliferation by reducing the expression of RelB. Immunohistochemical staining of CK19 and Ki67, as well as measurement of Ck19 mRNA levels in hepatic tissue, revealed that TP inhibited the BDL-induced ductular reaction. Masson 3 and Sirius Red staining for hepatic hydroxyproline showed that TP alleviated BDL-induced hepatic fibrosis. Additionally, TP substantially inhibited BDL-induced hepatic inflammation. In summary, TP inhibited the BDL-induced ductular reaction by reducing the expression of RelB in cholangiocytes, thereby alleviating liver injury, fibrosis, and inflammation.

## Introduction

Cholestasis can reflect either a functional defect in bile formation at the hepatocyte level or impairment in bile secretion/flow at the bile duct level (1). Cholestasis manifests as the excessive accumulation of biliary components (e.g., bile acid, cholesterol, and bilirubin) in the liver and systemic circulation. The clinical symptoms include liver injury, severe pruritus, jaundice, and fatigue; severe cases can cause acute liver failure (2). Chronic cholestasis may eventually lead to liver fibrosis and cirrhosis (3).

Ursodeoxycholic acid and chenodeoxycholic acid are the preferred drugs for treatment of cholestasis (4). However, tolerance may develop (5). Recently, obeticholic acid (a 6-ethyl derivative of chenodeoxycholic acid) was approved for the treatment of primary biliary cirrhosis—in conjunction with ursodeoxycholic acid—in patients with inadequate responses to ursodeoxycholic acid; it was approved as monotherapy for patients who are unable to tolerate ursodeoxycholic acid (6–8). However, hepatic decompensation, liver failure, and death have been reported in patients with Child-Pugh B or C cirrhosis who receive doses of obeticholic acid above the recommended level. Thus, the Food and Drug Administration placed a black box warning on the obeticholic acid label for patients with decompensated liver disease. New, inexpensive therapeutic agents are needed for effective relief of cholestasis symptoms.

Traditional Chinese medicine dietary supplements alleviate various forms of liver injury including cholestasis (9–12). *Tripterygium wilfordii* Hook.f (TWHF) is a species of Tripterygium in the Euonymus family (13); the dried root (“thunder god vine”) serves as a “bitter and cold” traditional Chinese medicine (14) in Eastern and Southern China. The root is also cooked in southern China. Triptolide (TP) is an epoxy diterpene lactone of TWHF (15), as well as the principal active ingredient in TWHF (13). TP exhibits potent immunosuppressive and antiproliferative activities (16); it effectively treats rheumatoid arthritis (17), diabetic kidney disease (18), and prostate cancer (19, 20). A TP dietary supplement reportedly alleviates senile osteoporosis (21), reduces stress, and increases longevity (22). We previously showed that TP was active against colon cancer (23, 24). The NF-κB protein complex regulates cell survival (25), aging (26), cytokine production (27), and obesity (28); NF-κB is the principal target of TP. NF-κB transcriptional inhibition by TP can suppress inflammation (29) and tumor growth (30). Thus far, the effects of TP on cholestasis remain unknown. In this study, we used a mouse model of common bile duct ligation (BDL) to explore whether TP can effectively treat cholestasis. Our findings provide a rationale for TP as complementary medicine of the preferred drugs or alternative medicine for cholestasis.

## Materials and methods

### Materials

Triptolide (CAS number 38748-32-2, purity > 98%) was purchased from Sanling Biotech (Guilin, China). TNFSF14 Elisa Kit (CSB-EL023991MO) was purchased from Cusabo (China).

Primary antibodies against RelB (10544), α-SMA (19245) and F4/80 (70076) was purchased from Cell Signaling Technology (USA). Primary antibody against CK19 (TROMA-III) was purchased from DSHB (USA). Primary antibody against GAPDH (60004-1-Ig) was purchased from Proteintech (China). Primary antibody against Ki67 (ab16667) was purchased form Abcam (USA). Primary antibody against Ly6g (4-5931-82) was purchased from Thermofisher (USA). Rabbit and Mouse secondary antibody (31460, 31430) were purchased from Thermofisher (USA). Rat secondary antibody (GB23302) were purchased from Servicbio (China).

### Animal surgery procedure

**FIGURE 1:**
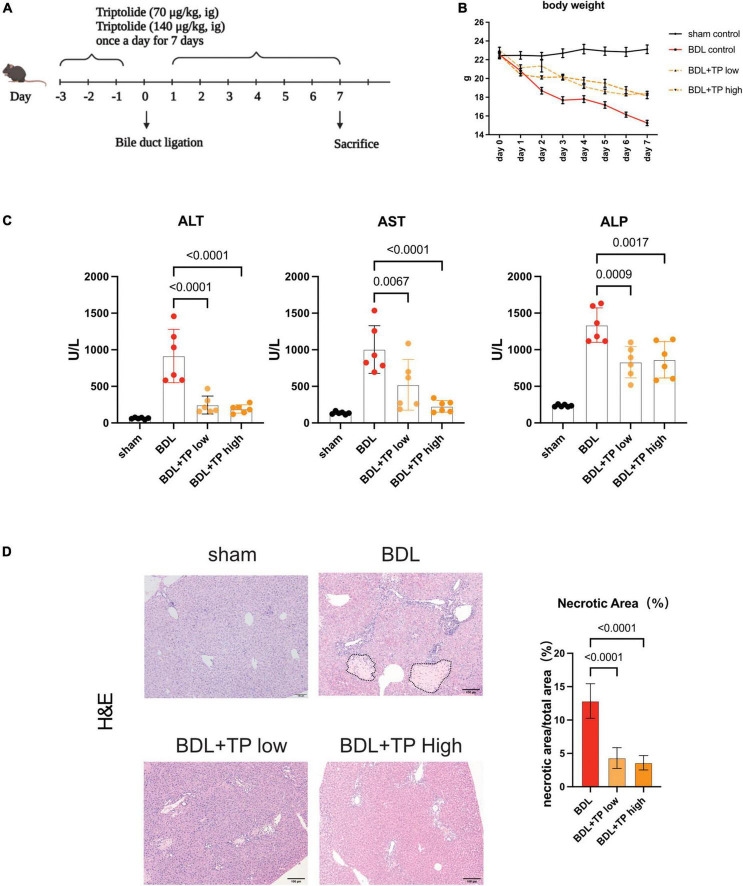
Triptolide alleviates liver injury induced by bile duct ligation. Male C57BL/6 mice were sacrificed at seven days after BDL or sham surgery. **(A)** The diagrammatic experimental procedures. **(B)** Body weight of mice (each group *n* = 6). **(C)** Serum levels of ALT, AST and ALP in mice sacrificed at 7 days after BDL or sham surgery. **(D)** Representative images of H&E (The black dotted line indicates the necrotic area) from liver tissues. Necrosis area statistics of H&E. Scale Bar: 100μm. Data are shown as the mean ± SD. Data represent at least 6 independent experiments with triplicate measurements. Analysis of variance (one-way ANOVA) was used. *p* values represents significance different from BDL group.

Male C57BL/6J mice (6–8 weeks) were supplied by Shanghai SLAC Laboratory Animal Co., Ltd (Shanghai, China). The animal study was reviewed and approved by the China Pharmaceutical University Experimental Animal Ethics Committee. Mice were housed in conditions with controlled light (12 h light/dark cycle), temperature (24 ± 2°C), and humidity (50–60%) and had adequate food and tap water. Cholestasis was induced by common bile duct ligation (BDL). Mice were anesthetized using 3% isoflurane and kept under anesthesia using 2–3% isoflurane during the entire infection procedure, where the abdominal cavity was opened from the abdominal midline. All experiments on mice were performed under the guidelines of Ethical Committee of China Pharmaceutical University. Triptolide in powder was suspended in 0.5% CMC-Na and administered to mice by gavage. The doses selected for TP in animal experiments were 70 μg/kg and 140 μg/kg (31). The common bile duct was ligated twice with a 7-0 nylon suture. The sham operation group involved the same operation, but the common bile duct was not ligated. After one-week acclimatization, the mice were then randomly separated into four groups (*n* = 6 per group): (1) Control mice (sham operated); (2) BDL mice; (3) BDL with TP at 70 μg/kg administration; (4) BDL with TP at 140 μg/kg administration. BDL performed at three days after TP treatment. Mice in sham and BDL group were given corresponding vehicle. After BDL the mice still were treated with TP once a day. Seven days after BDL surgery, mice were sacrificed (Figure 1A).

Serum alanine aminotransferase (ALT) and aspartate aminotransferase (AST) levels were measured using kits from Whitman Biotech (Nanjing, China). Hepatic hydroxyproline was measured using kits from Nanjing Jiancheng Bioengineering Institute (Nanjing, China). All kits were used according to the manufacturer’s protocols. Fragments of mouse livers were fixed overnight in buffered formaldehyde (10%) and embedded in paraffin for immunohistochemistry (IHC), hematoxylin and eosin (H&E), Masson’s trichrome and Sirius Red. H&E staining of liver tissue was carried out to observe pathological changes. All slides were scanned with a NanoZoomer S60 (Hamamatsu, Japan).

### Cell culture

Human intrahepatic biliary epithelial cells (HiBEC) were purchased from ScienCell and cultured in a EpiCAM (ScienCell) (Zhongqiaoxinzhou) containing 2% fetal bovine serum (FBS), EpiCGs (ScienCell), 5 μg/mL insulin and 0.5 μM hydrocortisone.

Small interference RNAs (siRNA) were purchased from GenePharma (Shanghai). The siRNA sequences used in this study are as follows: RelB-1 siRNA sense: 5′-GCCCGUCUAUGACAAGAAATT-3′; antisense: 5′-UUUCUUGUCAUAGACGGGCTT3′. RelB-2 siRNA sense: 5′-GCACAGAUGAAUUGGAGAUTT-3′, antisense: 5′-AUCUCCAAUUCAUCUGUGCTT-3′, Negative control siRNA: 5′-UUCUCCGAACGUGUCACGUTT-3′. HiBECs were seeded in six-well plate one day before transfection. HiBECs were transfected using Lipofectamine 3000 transfection kit (thermofisher, USA) according to the manufacturer’s instructions. Transfected cells were used for the subsequent experiments 48 h after transfection.

The growth cure of HiBECs was measured with the cck-8 kit (vazyme, China) assay. HiBEC were seeded in 96-well plate (5000 cells per well). The plates were incubated in full EpiCAM. Cck-8 working fluid were added in the plate 100 μL per well at 24h, 48h, 72h, 96h. After 1 h incubation with cck8, OD450 was detected using a spectrophotometer (Multiskan MK3, Thermofisher, USA).

### Quantitative real-time polymerase chain reaction

RNA from tissues and cells was extracted with TRIzol (vazyme, China). The RNA concentration was determined using Nanodrop2000 Spectrophotometers (Thermo Scientific, USA). cDNA was generated using BIO-RAD MyCyclerThermal Cycler (BIO-RAD, USA) and the HighCapacity cDNA Reverse Kit. qPCR was performed using StepOnePlus (Applied Biosystems, USA) with specific primers (Table 1). Primers were purchased from Genescipt (China). Results were normalized using GAPDH as an internal control.

**TABLE 1:** Primer sequences used for RT-PCR analysis.

Gene	Forward primer (5′-3′)	Reverse primer (5′-3′)
mouse *Ccn2*	GGGCCTCTTCTGCGATTTC	ATCCAGGCAAGTGCATTGGTA
mouse *Gapdh*	CTTTGGCATTGTGGAAGGGC	CAGGGATGATGTTCTGGGCA
mouse *Acta2*	TGCTGACAGAGGCACCACTGAA	CAGTTGTACGTCCAGAGGCATAG
mouse *Col1a1*	CCTCAGGGTATTGCTGGACAAC	CAGAAGGACCTTGTTTGCCAGG
mouse *Tgf-*β*1*	GCCACTGCCCATCGTCTACT	CACTTGCAGGAGCGCACAAT
mouse *F4/80*	CGTGTTGTTGGTGGCACTGTGA	CCACATCAGTGTTCCAGGAGAC
mouse *Il-1*β	TGGACCTTCCAGGATGAGGACA	GTTCATCTCGGAGCCTGTAGTG
mouse *Krt19*	AATGGCGAGCTGGAGGTGAAGA	CTTGGAGTTGTCAATGGTGGCAC
mouse *Tnf- a*	GGTGCCTATGTCTCAGCCTCTT	GCCATAGAACTGATGAGAGGGAG
mouse *Relb*	GTTCTTGGACCACTTCCTGCCT	TAGGCAAAGCCATCGTCCAGGA
mouse *Tnfsf14*	GGAGACATAGTAGCTCATCTGCC	CCACCAATACCTATCAAGCTGGC
mouse *Lt*β	CCTGTTGTTGGCAGTGCCTATC	GACGGTTTGCTGTCATCCAGTC
Human *Relb*	TGTGGTGAGGATCTGCTTCCAG	TCGGCAAATCCGCAGCTCTGAT
Human *Tnfsf14*	GGTCTCTTGCTGTTGCTGATGG	TTGACCTCGTGAGACCTTCGCT
Human *Lt*β	GGTTTCAGAAGCTGCCAGAGGA	CGTCAGAAACGCCTGTTCCTTC
Human *Gapdh*	GTCTCCTCTGACTTCAACAGCG	ACCACCCTGTTGCTGTAGCCAA

### Immunoblot analysis

Protein content was analyzed by lysing tissues and cells with RIPA buffer containing protease inhibitors and the Bradford Protein Assay Kit. Western blot analysis was performed following a previously described method (32). Protein bands were detected with a Tanon 5200Muti (Tanon, China) using ECL reagents. The gray density of the protein bands was determined using ImageJ. All quantitative comparisons between samples were on the same gels/blots.

### Immunofluorescence

Cells on coverslips were fixed in 4% paraformaldehyde for 15 min, washed with PBS, and permeabilized in PBS with 1% Triton for 10 min. Cells were then incubated with 5% goat serum in PBS for 1 h at room temperature before being incubated with antibodies overnight at 4°C. The next day, cells on a round coverslip were washed three times with PBS and incubated for 1 h at room temperature with secondary Alexa antibodies and DAPI. Fluorescence images were scanned using a FV3000 (Olympus, Japan).

### Statistical analysis

All data were shown as mean ± SD and at least three replicate experiments were performed *in vitro* and *in vivo*. The necrotic, Masson3 positive and Sirius Red positive area were analyzed using Image J software. Statistical significance was determined using one-way analysis of variance as appropriate (GraphPad Prism 9, GraphPad Software Inc., CA).

## Results

### Triptolide alleviates bile duct ligation-induced liver injury

To explore the effects of TP on cholestasis-induced liver injury, we established a mouse model of BDL and administered two TP doses by oral gavage; such doses were previously reported to attenuate chronic kidney disease (31). A schematic of the mouse model is depicted in Figure 1A. Figure 1B shows that TP at a dose of 70 or 140 μg/kg attenuated the BDL-induced weight loss. BDL increased the serum levels of the liver enzymes alanine transaminase, aspartate aminotransferase, and alkaline phosphatase. Either dose of TP substantially reduced the levels of these enzymes (Figure 1C). Histopathological staining revealed less necrosis around the portal tract when BDL mice were treated with TP (Figure 1D). Thus, TP effectively treated BDL-induced liver injury.

### Triptolide inhibits proliferation and RelB expression in human intrahepatic biliary epithelial cells

Bile duct hyperplasia is common in patients with cholestasis; cholangiocyte proliferation and a ductular reaction contribute to the onset and progression of liver disease (32–34). Members of the NF-κB family of transcription factors act through a canonical pathway and a non-canonical pathway. Non-canonical NF-κB signaling activates predominantly p100-sequestered NF-kB proteins, the most important of which is RelB (35). This protein is involved in the ductular reaction; the bile ducts of patients with primary sclerosing cholangitis and primary biliary cirrhosis exhibit increased levels of RelB. RelB and its downstream target lymphotoxin β (LTβ) affect the proliferation of bile duct epithelial cells (36). TP inhibits the expression of NF-κB proteins (37). Here, we analyzed HiBECs *in vitro*. We hypothesized that TP would reduce cholangiocyte proliferation by inhibiting the expression of RelB.

**FIGURE 2:**
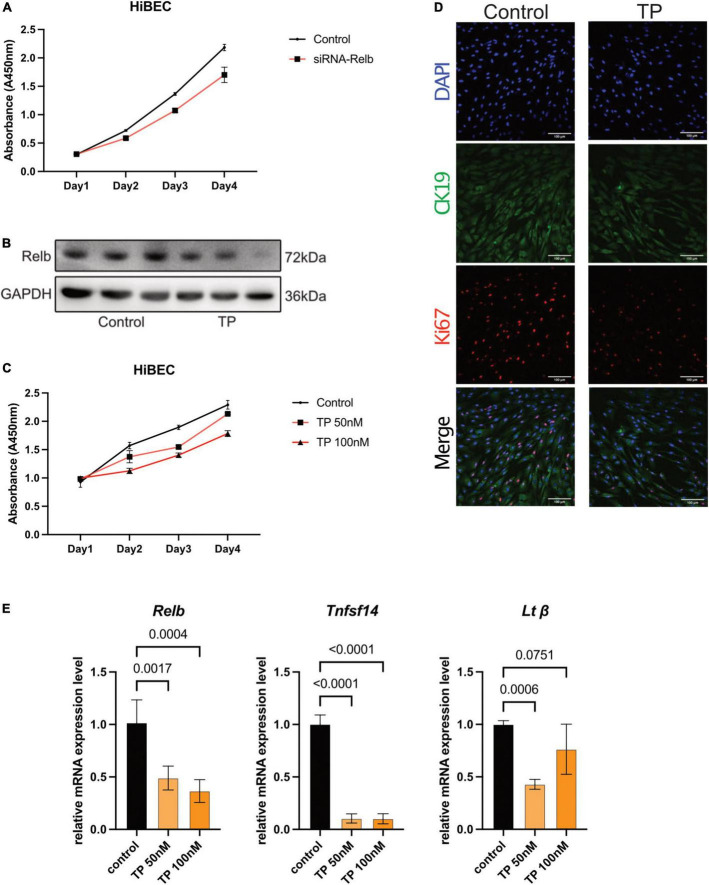
Triptolide inhibits proliferation and RelB expression in HiBEC. **(A)** The growth curve of HiBEC after transfection of siRNA-*Relb*. **(B)** The protein level of RelB in HiBEC after TP treatment. **(C)** The growth curve of HiBEC after TP treatment. **(D)** Double immunofluorescence staining for CK19 (green) and Ki67 (red) from HiBEC after TP treatment. Nuclei were counter-stained with DAPI (blue). **(E)** *Relb*, *Tnfsf14* and *Lt*β mRNA was measured in HiBEC after TP treatment. Data are shown as the mean ± SD. Data represent at least 3 independent experiments with triplicate measurements. Analysis of variance (one-way ANOVA) was used. *p* values represents significance different from control group.

Growth curve analyses showed that *siRNA*-mediated RelB knockdown inhibited the growth of HiBECs (Figure 2A). Western blotting revealed that TP (100 nM) significantly inhibited the expression of RelB in HiBECs (Figure 2B). TP at 50 and 100 nM inhibited the growth of HiBECs (Figure 2C); this was confirmed (for TP at 100 nM) by immunofluorescence staining of CK19 and Ki67 (proliferation markers) (Figure 2D). Quantitative polymerase chain reaction (qPCR) analysis demonstrated that TP significantly reduced the mRNA expression levels of *Relb* and the downstream genes *Tnfsf14* and *Lt*β (Figure 2E).

### Triptolide inhibits bile duct ligation-induced expression of RelB and downstream genes

**FIGURE 3:**
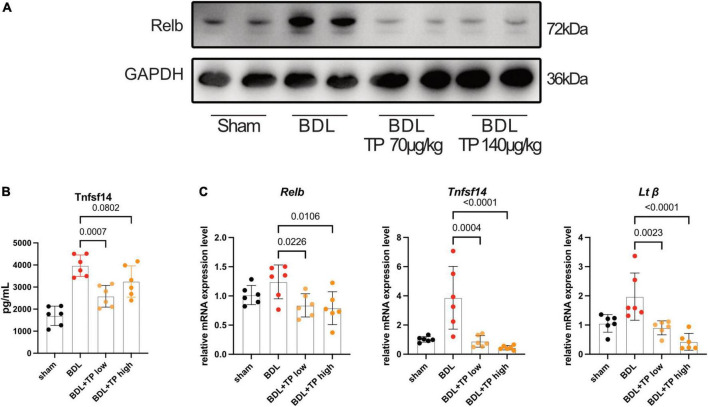
Triptolide inhibits BDL induced RelB and its downstream gene expression. **(A)** Western blot analysis of RelB in liver. **(B)** The serum TNFSF14 levels (ELISA data) of mice. **(C)** The mRNA level of *Relb*, *Tnfsf14* and *Lt*β in liver tissues. Data are shown as the mean ± SD. Data represent at least 6 independent experiments with triplicate measurements. Analysis of variance (one-way ANOVA) was used. *p* values represents significance different from BDL group.

Western blotting revealed that the protein level of RelB increased after BDL. Both TP doses substantially reduced the level of RelB (Figure 3A). Enzyme-linked immunosorbent assay analysis showed that BDL increased the expression of serum tumor necrosis factor superfamily member 14 (TNFSF14), whereas TP inhibited this increase (Figure 3B). qPCR analysis of hepatic tissue showed that BDL upregulated the mRNA expression levels of *Relb*, *Tnfsf14*, and *Lt*β, but these increases were inhibited by TP at a dose of 70 or 140 μg/kg (Figure 3C).

### Triptolide relieves bile duct ligation-induced bile duct hyperplasia

**FIGURE 4:**
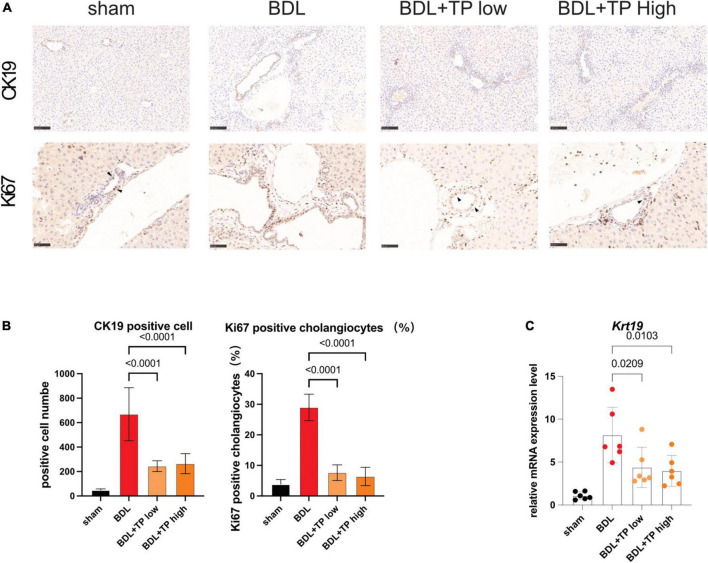
Triptolide relieves BDL-induced bile duct hyperplasia. **(A)** Representative images of IHC for CK19 and Ki67 (The black arrows indicate the regenerated cholangiocytes) from liver tissues. Scale Bar: 100 μm. **(B)** Statistical analysis of immunohistochemically positive regions of **(A)**. **(C)** The mRNA level of *Ck19* in liver tissues. Data are shown as the mean ± SD. Data represent at least 6 independent experiments with triplicate measurements. Analysis of variance (one-way ANOVA) was used. *p* values represents significance different from BDL group.

The above results indicated that TP inhibited the BDL-induced upregulation of *Relb* and downstream genes (*Tnfsf14* and *Lt*β) in hepatic tissue. Increased levels of RelB lead to a ductular reaction. Cytokeratin-19 (CK19) is solely expressed by cholangiocytes. Immunohistochemical analysis of CK19 revealed that BDL induced prominent bile duct hyperplasia; TP inhibited this process (Figures 4A,B). Immunohistochemical analysis of Ki67 revealed many positive cells (black arrows) in bile ducts after BDL; TP significantly reduced the numbers of these cells (Figures 4A,B), indicating that TP alleviated bile duct hyperplasia. qPCR analysis showed that TP significantly reduced the BDL-induced upregulation of *Ck19* (Figure 4C).

### Triptolide relieves bile duct ligation-induced liver fibrosis

**FIGURE 5:**
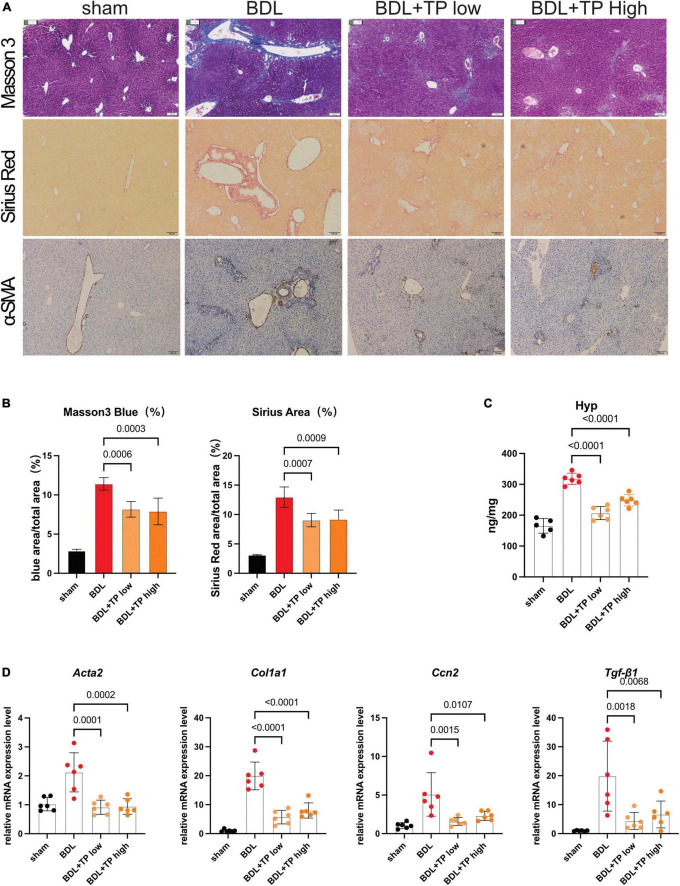
Triptolide relieves BDL-induced liver fibrosis. **(A)** Representative images of Masson 3, Sirius Red, and IHC for α-SMA from liver tissues. Scale Bar: 100 μm. **(B)** Collagen positive area statistics of Masson3 and Sirius Red. **(C)** Hydroxyproline assay of liver tissues. **(D)** The mRNA levels of *Acta2*, *Col1a1*, *Ccn2*, and *Tgf-*β*1* from liver tissues. Data represent at least 6 independent experiments with triplicate measurements. Analysis of variance (one-way ANOVA) was used. *p* values represents significance different from BDL group.

TNFSF14, which acts downstream of RelB, promotes hepatic stellate cell activation and exacerbates liver fibrosis (38). Staining with Masson 3 and Sirius Red confirmed that TP decreased collagen deposition around the portal fields in BDL mice (Figures 5A,B). α-Smooth muscle actin [also known as actin alpha 2 (ACTA2)] is a marker of hepatic stellate cell activation; immunohistochemical staining of α-smooth muscle actin decreased around the portal area (Figure 5A). Hydroxyproline is a characteristic component of collagen; in BDL mice, the hepatic levels of hydroxyproline were substantially lower after treatment with TP at a dose of 70 or 140 μg/kg, compared with those levels in the control group (Figure 5C). Next, we examined the expression of liver fibrosis-related genes. Col1a1 is an important collagen component, and its expression significantly increases in fibrotic tissues. Connective tissue growth factor [also known as cellular communication network factor 2 (CTGF/CCN2)] and transforming growth factor beta-1 (TGF-β1) are markers of liver fibrosis, and they both directly activate hepatic stellate cells and promote collagen deposition; BDL elevates the levels of both proteins (39, 40). The mRNA expression levels of *Acta2*, *Col1a1*, *Ccn2*, and *Tgf-*β*1* were downregulated when BDL mice were treated with TP at a dose of 70 or 140 μg/kg (Figure 5D).

### Triptolide relieves bile duct ligation-induced hepatic inflammation

**FIGURE 6:**
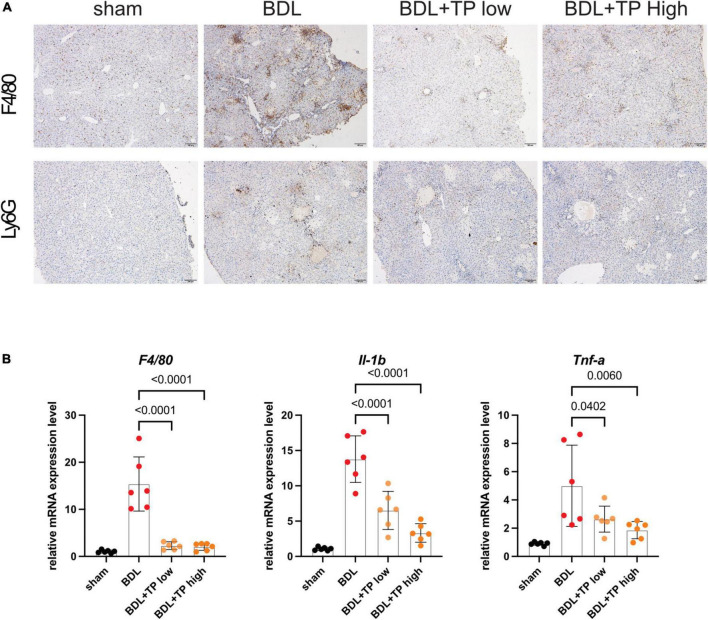
Triptolide relieves BDL-induced liver inflammation. **(A)** Representative images of IHC of F4/80 and LY6G from liver tissues. Scale Bar: 100 μm. **(B)** The liver mRNA levels of *F4/80*, *IL-1*β and *Tnf-*α from liver tissues. Data represent at least 6 independent experiments with triplicate measurements. Analysis of variance (one-way ANOVA) was used. *p* values represents significance different from BDL group.

Ductular reactions are often accompanied by inflammatory infiltrates (33, 41). Therefore, we examined the effect of TP on hepatic inflammation. For this purpose, we conducted immunohistochemical staining of F4/80 (also known as mouse EGF-like module-containing mucin-like hormone receptor-like 1), which is expressed by various mature macrophages including Kupffer cells; we also performed immunohistochemical staining of the lymphocyte antigen 6 complex locus G6D (LY6G), a neutrophil-specific marker. BDL-induced enhancement of F4/80 and LY6G staining was decreased by TP at a dose of 70 or 140 μg/kg; thus, TP reduced hepatic inflammatory infiltration (Figure 6A). The mRNA expression levels of genes encoding the inflammatory factors F4/80, interleukin-1β, and tumor necrosis factor-α were significantly reduced when BDL mice received TP at a dose of 70 or 140 μg/kg (Figure 6B). These findings indicated that TP attenuated hepatic inflammatory infiltration in BDL mice.

## Discussion

**FIGURE 7:**
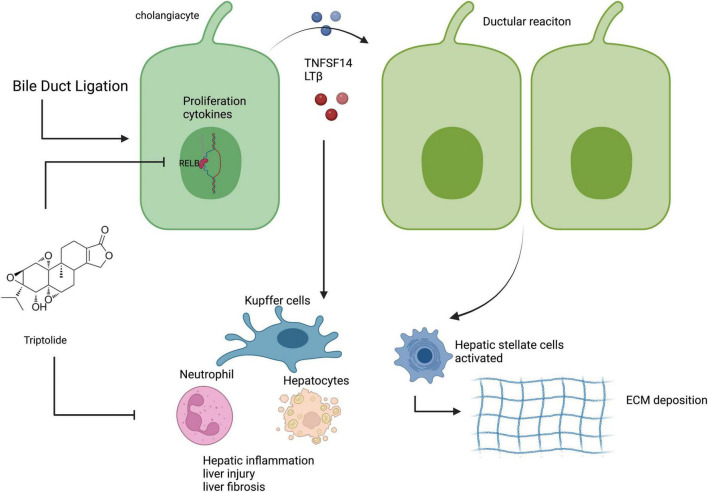
TP inhibited the BDL-induced ductular reaction by reducing the expression of RelB in cholangiocytes, thereby alleviating liver injury, fibrosis, and inflammation. Created with BioRender.com.

We explored whether TP protected against liver injury progression in a mouse model of common BDL. TP at a dose of 70 or 140 μg/kg effectively treated BDL-induced liver injury. Liver enzyme measurement and H&E staining revealed that TP at a dose of 70 or 140 μg/kg significantly alleviated liver damage. Analysis of the liver hydroxyproline content, along with Masson 3 and Sirius Red staining, revealed that TP inhibited BDL-induced liver fibrosis. qPCR analysis of *Ck19* transcripts, as well as immunohistochemical staining of CK19 and Ki67, showed that TP significantly inhibited the BDL-induced ductular reaction. TP substantially reduced hepatic inflammatory infiltration after BDL, as revealed by immunohistochemical staining of F4/80 and Ly6G, as well as the mRNA expression levels of *F4/80, Il-1*β, and *Tnf-*α in hepatic tissue. *In vitro* analysis demonstrated that TP dramatically downregulated the protein and mRNA expression levels of RelB, as well as the downstream genes *Tnfsf14* and *Lt*β, thereby slowing the growth of HiBECs. Assessment of protein and mRNA expression levels in hepatic tissue revealed that TP attenuated the BDL-induced upregulation of RelB and downstream genes. The serum TNFSF14 assay confirmed that TP alleviated the BDL-induced upregulation of RelB. Graphic abstract was shown in Figure 7.

*Tripterygium wilfordii* Hook.f (TWHF) exhibits anti-inflammatory (41), anti-fertility (42), anti-colitis (43), and anti-cancer activities (44). At present, the clinical medication of TWHF is mainly used for rheumatoid arthritis, lupus and purpuric nephritis, psoriasis, erythroderma and allergic diseases. There are no clinical trials linking TWHF with cholestatic disease. However, the therapeutic window for TP is narrow; clinical applications are compromised by severe toxicities, including hepatotoxicity (37). The doses in this study were chosen because TP at a dose of 70 μg/kg substantially alleviated chronic kidney disease (31); we also used a higher dose for comparison. In our previous study, it was found that there was no obvious liver toxicity and cholestasis symptoms when TP 250 μg/kg was administered for 7 days (45). Therefore, we believe that the TP dose used in this study is a safe dose. TP-induced hepatotoxicity cannot be ignored. We previously found that TP was hepatotoxic at a dose of 500 or 600 μg/kg (46, 47). Evidently, TP at a dose of 70 or 140 μg/kg significantly alleviated BDL-induced liver injury, liver fibrosis, the ductular reaction, and hepatic inflammatory infiltration. Therefore, the role of TP in cholestasis is dose-dependent. The specific mechanism may be related to the complex immune homeostasis in the liver, and the specific mechanism will be carried out in future studies. The above content shows that TP needs more research before clinical treatment of cholestasis and may require structural modification and more accurate individualized diagnosis and treatment strategies.

Cholestasis can be caused by certain drugs, abnormal hormone levels, hepatitis, and dietary habits, but the underlying mechanism remains unknown (48). Recent studies have shown that the ductular reaction plays an important role in cholestasis-induced liver fibrosis and injury. The ductular reaction, characterized by cholangiocyte hyperproliferation, is commonly observed in patients with biliary disorders such as primary biliary cirrhosis, primary sclerosing cholangitis, or biliary atresia; this reaction is usually associated with liver fibrosis, and the extent of fibrosis is often correlated with mortality (32, 34). We presume that the reaction reflects the intense local inflammatory microenvironment present in cholangiocellular cholestasis; damaged cholangiocytes proliferate to compensate for reduced biliary cell function. The ductular reaction exacerbates hepatic inflammation, inhibits liver regeneration, and promotes fibrosis (41, 49–52). Previous studies showed that inhibition of the ductular reaction alleviated cholestasis-induced liver damage, inflammation, and fibrosis (53, 54).

Some authors reported that the level of RelB was directly related to the extent of ductular reaction. RelB and LTβ were highly expressed in cholangiocytes from patients with chronic liver diseases (hepatitis C and hepatitis B virus infections, alcoholic liver disease, non-alcoholic fatty liver disease, and autoimmune hepatitis) or cholangiopathies (primary biliary cirrhosis and primary sclerosing cholangitis) (36). The activation of RelB in cholangiocytes and hepatocytes induces the secretion of LTβ, which activates cholangiocyte RelB in both an autocrine and paracrine manner through the LTβ receptor, thereby stimulating bile duct proliferation. Thus, RelB is essential for cholangiocyte proliferation and the ductular reaction. But so far, there are no drugs targeting RelB yet in clinical, therefore, the design of new drugs for RelB has broad prospects. Accordingly, we first examined the effect of TP on cholangiocyte proliferation *in vitro*. As expected (37), TP (an NF-κB inhibitor) significantly reduced cell proliferation, as well as the expression of RelB and its downstream genes. *In vivo* analysis showed that TP significantly alleviated the BDL-induced upregulation of RelB. This finding suggested that TP reduces cholangiocyte expression of RelB, thus suppressing cholangiocyte proliferation; alleviating the BDL-induced ductular reaction; and reducing liver damage, inflammation, and fibrosis.

RelB is frequently associated with liver fibrosis. In addition to its presence in cholangiocytes, RelB is expressed in Kupffer cells (55) and hepatocytes (36). RelB-regulated TNFSF14 is presumed to promote hepatic stellate cell activation and the resulting liver fibrosis (38). Our assays of serum TNFSF14 levels indicated that TP reduced the BDL-induced increase in the TNFSF14 level; this may partly explain why TP relieves hepatic inflammation and fibrosis.

Finally, TP is a small-molecule drug and may thus act via several mechanisms. We focused on RelB. More detailed clinical and translational studies are needed to substantiate the potential utility of TP as a cholestasis treatment. Careful dosing studies are also essential.

## Conclusion

Triptolide (TP) at certain doses improved cholestasis. TP may be useful in the prevention or treatment of cholestasis-induced liver injuries, fibrosis, and other inflammatory diseases.

## Data availability statement

The original contributions presented in this study are included in the article/Supplementary material, further inquiries can be directed to the corresponding authors.

## Ethics statement

The animal study was reviewed and approved by China Pharmaceutical University Experimental Animal Ethics Committee.

## Author contributions

ZJ and ZhY designed the overall research experiments. ZhY, JW, HZ, YM, QT, ZqY, and CN performed the experiments. ZhY and JW analyzed the data. ZhY wrote the manuscript. ZJ and QY reviewed the manuscript. All authors contributed to the article and approved the submitted version.
