# Supplementary material for: Triptolide increases resistance to bile duct ligation-induced liver injury and fibrosis in mice by inhibiting RELB
Source: Front Nutr. 2022 Oct 13;9:1032722. doi: 10.3389/fnut.2022.1032722 (PMC9608656; doi:10.3389/fnut.2022.1032722)
Supplement: Supplementary file 1 [file Data_Sheet_1.ZIP › raw data/Original data.docx]

The original data of IHC, H&E, Masson 3, Sirius were uploaded in icloud. Please refer to the link blow:

https://www.jianguoyun.com/p/DT0p8c4QiO7xChiy79QEIAA
