# Supplementary figures and images for: Triptolide increases resistance to bile duct ligation-induced liver injury and fibrosis in mice by inhibiting RELB
Source: Front Nutr. 2022 Oct 13;9:1032722. doi: 10.3389/fnut.2022.1032722 (PMC9608656; doi:10.3389/fnut.2022.1032722)

Figure 2.B

RELB

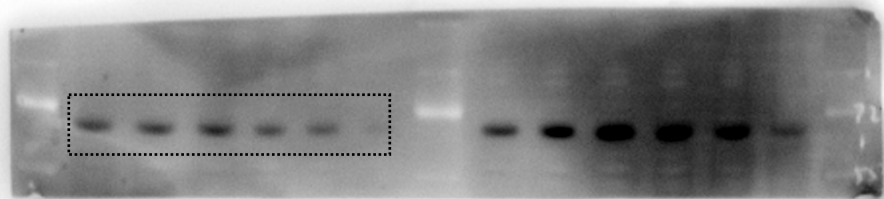

GAPDH

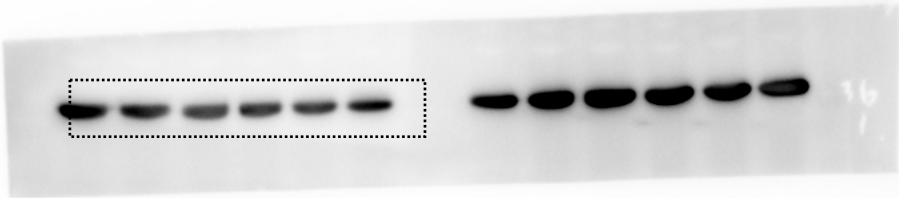

Figure 3.A

RELB

GAPDH

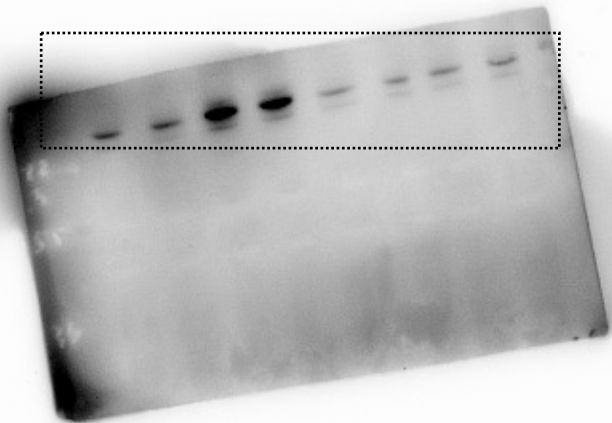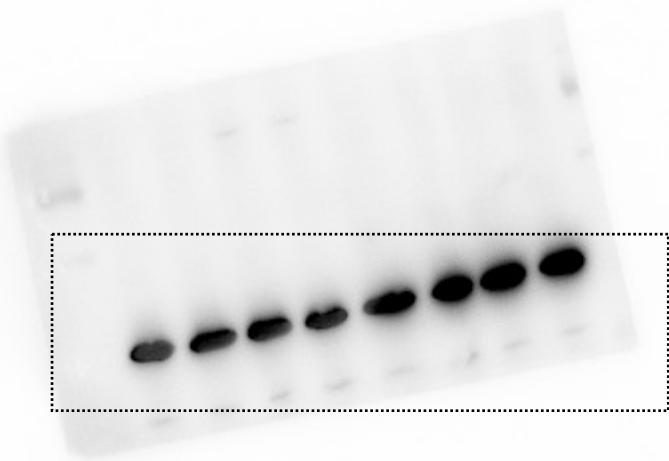

Supplement: Supplementary file 1 [file Data_Sheet_1.ZIP › raw data/WB raw data.pdf]
